# Distribution of lymph node metastasis from lymphoepithelial-like carcinoma of the parotid

**DOI:** 10.18632/oncotarget.11002

**Published:** 2016-08-02

**Authors:** LiNa Yin, Xue Huang, XiaoLan Liu, YongChun Zhang, Xiaoshen Wang

**Affiliations:** ^1^ Department of Radiation Oncology, Fudan University Shanghai Cancer Center, and Department of Oncology, Shanghai Medical College of Fudan University, China; ^2^ Department of Radiation Oncology, Changzhou Tumor Hospital, Soochow University, Changzhou, China; ^3^ Department of Radiation Oncology, The Second Affiliated Hospital of Jilin University, Changchun, China; ^4^ Department of Radiation Oncology, The Affiliated Hospital of Qingdao Medical College, Qingdao University, Qingdao, China

**Keywords:** lymphoepithelial-like carcinoma (LELC), parotid gland, cervical lymph nodes

## Abstract

**Purpose:**

To explore the distribution of node spread from lymphoepithelial-like carcinoma (LELC) of the parotid gland based on the 2013 updated guidelines for neck node levels.

**Results:**

42 (58.3%) cases had metastatic nodes, all were localized at the ipsilateral neck. The detailed distribution was: level Ia 0, level Ib 6(14.3%), level II 34 (80.1%), level III 16 (38.1%), level IV 9 (21.4%), level V 7 (16.7%), level VI 0, level VII 0, level VIII 37 (88.1%), level IX 0, level Xa 2 (4.8%), and level Xb 0. Lymphadenopathy in level Ib, V and Xa was always accompanied with level II or level VIII nodal metastasis. No statistical significance was found in the incidence of nodal involvement between T1-2 and T3-4 tumors (57.4% versus 61.1%, *p* = 0.78).

**Methods:**

We retrospectively reviewed the surgical and imaging documents of 72 cases of LELC from the parotid gland between January 2004 and November 2015. All patients received contrast enhanced computed tomography (CT) or magnetic resonance imaging (MRI). Parotid metastasis from nasopharyngeal cancer (NPC) was excluded. Nodal status and distribution was evaluated by both pathologic reports and imaging studies.

**Conclusions:**

This is the first description of topography of cervical nodal metastases from LELC of the parotid gland. Incidence of nodal involvement is high. Nodes at ipsilateral level VIII and II were most frequently involved, followed by level III, IV, V and Ib. Nodes in level Ia, VI and level VII were rarely seen.

## INTRODUCTION

Lymphoepithelial-like carcinoma (LELC) of the salivary gland is a rare malignancy. It was first described by Hilderman et al. in 1962 [1]. The World Health Organization (WHO) has defined it as ‘‘a poorly differentiated squamous cell carcinoma or histologically undifferentiated carcinoma accompanied by a prominent reactive lymphoplasmacytic infiltrate, morphologically similar to undifferentiated nasopharyngeal carcinoma (NPC)’’ [2]. But LELC is separated from NPC by its location and clinical outcome. LELC has been diagnosed in numerous organs of the head and neck region. But the majority (about 80%) is located in the parotid gland [3, 4].

LELC of the parotid gland is distinctly more common in certain ethnic groups such as Eskimos/Inuits from Alaska, Canada, Greenland, Japanese, and Southeastern Chinese [5–7].

The standard treatment for LELC of the parotid gland remains controversial. Some researchers recommend multimodality therapy including surgical resection of the primary lesion combined with neck dissection, followed by radiation therapy with or without chemotherapy [8]. The published literature showed that about 40%–77% of LELC patients presented with regional lymph node metastasis [4, 9–15], thereby necessitating routine neck dissection. However, unlike other high-grade salivary gland carcinomas, LELC is very radiosensitive [12–15]. Therefore, radiotherapy has been used instead of surgery as the primary treatment of LELC [12]. One report from the University of Texas M. D. Anderson Cancer Center indicated that the 5-year actuarial local control rate for LELC was 94% treated with radiotherapy. The main cause of treatment failure was distant metastasis. The 5-year actuarial rate of distant metastasis was 30% [12]. Another report from Hongkong also suggested that the prognosis of LELC treated by radiotherapy was good: the 5-year control rate exceeded 90% and the 4-year disease-free survival rate reached 85.7% [13].

Regardless of surgery or radiotherapy, an understanding of the pattern of nodal distribution might provide useful information for individulized treatment of the neck [16]. However, due to the rarity of this malignancy, report on topography of nodal disease from LELC was scarce. Hence, there is no consensus as to which nodal levels should be included in a prophylactic neck dissection or extent of therapeutic neck dissection. Knowledge regarding which nodal levels should be contoured as the clinical target volume (CTV) is also lacking in curative radiotherapy or post-operative setting.

Therefore, we carried out this retrospective study to analyze the patterns of nodal metastasis from LELC of the parotid gland.

## RESULTS

### Incidence and distribution of nodal metastasis

Of the 72 patients with LELC of the parotid gland, 42 (58.3%) had positive lymph nodes. The incidence of nodal involvement in T1-2 disease was 57.4% (31 out of 54), and 61.1% (11out of 18) in T3-4 lesion, respectively (*p* = 0.78). There was no contralateral or bilateral nodal disease. All metastatic nodes were located in the ipsilateral neck. The detailed distribution was shown in Table 1.

**Table 1:** Topography of nodal disease in 42 patients with positive node

Nodes	Ipsilateral
Level Ia	0
Level Ib	6(14.3%)
Level II	34 (80.1%)
Level III	16 (38.1%)
Level IV	9 (21.4%)
Level V	7 (16.7%)
Level VI	0
Level VII	0
Level VIII	37 (88.1%)
Level IX	0
Level Xa	2(4.8%)
Level Xb	0

**Figure 1:**
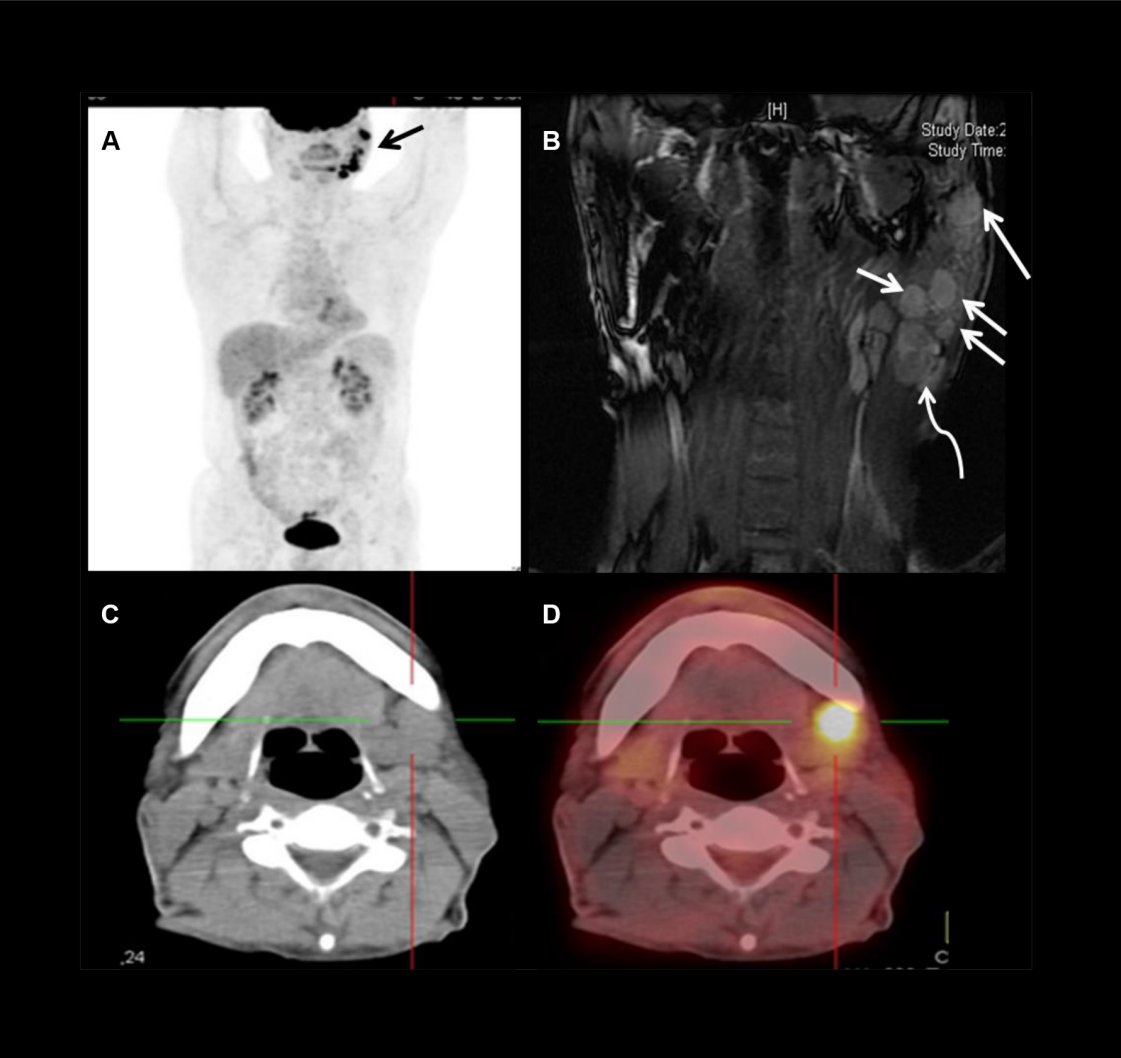
Images showing level Ib and level VIII nodes metastases in a 58 years old male patient with LELC of the left parotid gland (**A**) PET panorama demonstrated multiple nodules (arrow) in the left parotid and upper neck with high uptake of FDG. (**B**) Coronal MRI showed multiple metastases (arrows) in and just beneath (curved arrow) the left parotid. (**C** and **D**) CT-PET suggested level Ib nodal involvement.

Level I: No solitary metastasis to level Ib was found. Of the 6 patients with level Ib node involvement (Figure 1C and 1D), all were accompanied with both level II and level VIII lymphadenopathy. But none had nodal disease at level Ia.

Level II: Since the final histopathologic reports did not subdivide level II nodes into level IIa or IIb, consequently, we only analyzed the total number of level II lymphadenopathy. Altogether, 34 (80.1%) cases had positive nodes in level II.

Level III: 16 (38.1%) cases were with level III node involvement, all were located laterally or posteriorly to the carotid sheath, accompanied with level II or level VIII nodal disease.

Level IV: Positive nodes in level IV were found in 9 (21.4%) cases, all accompanied with level II lymphadenopathy, and all were located laterally or posteriorly to the carotid sheath.

Level V: 7 (16.7%) cases had nodal disease in level V. All patients were simultaneously accompanied with nodal involvements in both level II and VIII.

Level VIII: The highest incidence of nodal metastases was in level VIII, 37 (88.1%) patients had positive nodes in this area (Figure 1A and 1B). Metastases in this area could be solitary, or multiple, or accompanied with lymphadenopathy in other levels.

Level X: Metastasis to level X was rare, only 2 patients had nodal disease in level Xa, and were accompanied with extensive ipsilateral lymphadenopathy.

**Figure 2:**
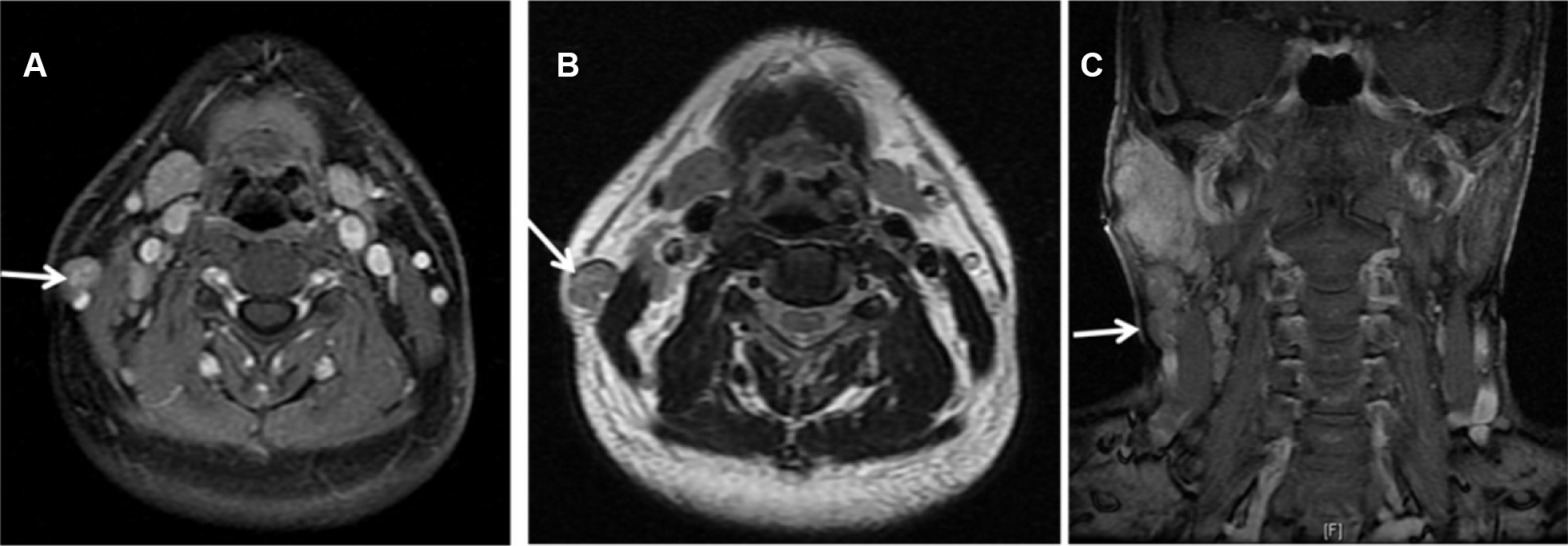
MRI showing nodal metastasis (arrow) at the surface of the right sternocleidomastoid muscle in a 51 years old male patients with LELC of the right parotid (**A**) Contrast enhanced transverse T1WI MRI with fat suppression. (**B**) Transverse T2WI MRI. (**C**) Contrast enhanced coronal T1WI MRI with fat suppression.

There were 5 patients with nodal metastasis located between the lateral border of the sternocleidomastoid muscle and the platysma (Figure 2), much lower to the parotid gland, but lateral to level II node. From the anatomic point of view, they did not belong to any of the levels classified in the 2013 updated consensus guidelines. These 5 patients had multiple nodal involvements at the ipsilateral neck.

## DISCUSSION

LELC is morphologically similar to undifferentiated NPC. The parotid gland is the most common location for LELC to arise [3, 4]. Parotid gland LELC has been reported to occur almost exclusively in Greenland Eskimo, North American Eskimo, and Chinese patients [5–7, 12]. Worldwide, LELC of the parotid gland is still rare.

Up to date, there is no standard treatment for LELC of the parotid. Since LELC has a high propensity for harboring nodal metastasis, with 40%–77% patients having enlarged nodes at diagnosis [9–15], and our study showed that 58.3% patients had nodal involvement. Some authors recommend surgical resection of both the primary lesion and neck node, followed by radiation therapy with or without chemotherapy [8]. However, due to its pathologic characteristics, LELC is very sensitive to both radiation and chemotherapy [12–15]. Therefore, some authors recommend radiotherapy as the primary treatment of LELC [12]. Reports from the University of Texas M. D. Anderson Cancer Center and Hongkong both indicated that the local control rate for LELC exceeded 90% treated with radiotherapy. The main cause of treatment failure was distant metastasis [12, 13].

Regardless of surgery or radiotherapy, the knowledge regarding the patterns of node spread from LELC will be of great help not only for surgeons when performing neck dissection, but also for radiation oncologists when delineating the target volume. Our study demonstrated that nodes at ipsilateral level VIII and II were most frequently involved, followed by level III, IV, Ib and V. Nodes in level Ia, VI and level VII were rarely seen. Based on literature report, the lymphatic drainage for each organ uses several pathways including the main collecting way and alternative routes [17]. The alternative routes should be included in the target volume definition in dependence of the feasibility for that route [18]. Almost all of the lymph from the parotid gland is drained into the superficial and profound parotid lymph nodes (level VIII). The efferent lymph collectors of the parotid lymph nodes transport the lymph further into the upper third of the internal jugular lymph nodes (level II), and then to the middle third (level III), followed by the lower third (level IV). In rare cases, a lymph collector passes from the frontal lower section of the parotid gland through the masseter muscle to the submandibular lymph nodes (level Ib). Drainage of the posterior section of the parotid gland into the accessory chain (level V) is equally rare [19]. The topography of nodal distribution as demonstrated in our study was in good accordance to the above mentioned lymphatic drainage pathway of the parotid. Another drainage pathway should also be taken into account. Since the posterior part of the parotid gland was located at the surface of the sternocleidomastoid muscle, the efferent lymph collectors of this part might transport the lymph down through the space between the lateral border of the sternocleidomastoid muscle and the platysma. As demonstrated in our study, 5 patients had nodal disease in this space.

For undifferentiated NPC, the incidence of nodal involvement seemed to have no correlation with T stage [20]. Regarding LELE of the parotid, our study demonstrated that there was no statistical significance in the incidence of nodal involvement between T1-2 and T3-4 tumors (57.4% versus 61.1%, *p* = 0.78). This suggested that the extent of neck dissection or the volume of prophylactic neck irradiation should not depend on the size of the primary lesion.

## MATERIALS AND METHODS

### Patients

We retrospectively reviewed the medical documents of the patients with malignant tumors of the parotid gland treated between January 2004 and November 2015, and identified 81 cases of pathologically confirmed LELC. However, only 72 cases had comprehensive surgical and imaging records, and were enrolled in our study. The clinical characteristics of the 72 patients with LELC of the parotid gland were shown in Table 2.

**Table 2:** Clinical characteristics of the 72 patients with LELC

Median age (years)	46 (range:15–78)
Gender	
Male	42 (58.3%)
Female	30 (41.7%)
Imaging tools	
CT	25 (34.7%)
MRI	47 (65.3%)
T stage	
T1-2	54 (75%)
T3-4	18 (25%)
Node status	
N0	30 (41.7 %)
N+	42 (58.3%)
Surgery modality	
Biopsy only	6 (8.3%)
PLR only	21 (29.2 %)
PLR+ UUND	31 (43.1 %)
PLR+ UCND	14 (19.4%)

Abbreviations: CT = computed tomography, MRI = magnetic resonance imaging, PLR = primary lesion resection, UUND = unilateral upper neck dissection, UCND = unilateral comprehensive neck dissection.

### Diagnosis of LELC

Since LELC of the parotid gland and NPC are co-prevalent in China, and LELC is similar with non-keratinizing undifferentiated NPC in several aspects, such as its relationship with EBV, its histomorphology, and the predilection of both malignances for certain regions and populations. It is therefore necessary to distinguish LELC and parotid metastasis from NPC.

Before making the final diagnosis of LELC, all patients underwent comprehensive physical examination, fibreoptic nasopharyngoscopy, pre and post contrast magnetic resonance imaging (MRI), or contrast enhanced computed tomography (CT). Any imaging abnormalities of the nasopharynx received biopsy at least twice in order to exclude NPC.

### Imaging studies

25 patients received contrast enhanced CT of the parotid and whole neck, the section thickness was 5 mm without interslice gap for the axial plane. 47 patients underwent pre and post contrast MRI. The examined area extended from the anterior clinoid process to the inferior margin of the clavicle. T1-weighted fast spin-echo images (repetition time [TR] 400–500 ms and echo time [TE] 10–15 ms), and T2-weighted fast spin-echo images in the axial planes (TR 4000–5000 ms and TE 80–100 ms) were obtained before injection of contrast material. After intravenous injection of gadolinium-complexed diethylene triamine penta-acetic acid, T1-weighted fast spoiled gradient echo fat-suppressed axial and coronal sequences were acquired (TR 150–250 ms and TE 2–10 ms). Section thickness was 6 mm with a 1-mm interslice gap for the axial plane, and 4 mm without interslice gap for the coronal planes. In addition to CT or MRI, 14 patients also received whole body 18F-deoxyglucose positron emission tomography-computed tomography (PET-CT).

### Surgical procedure

Of the seventy-two patients with LELC of the parotid gland, six (8.3%) received biopsy only, twenty-one (29.2%) primary lesion resection (PLR), thirty-one (43.1%) PLR combined with unilateral upper neck dissection (UUND), and fourteen (19.4%) PLR combined with unilateral comprehensive neck dissection (UCND).

### Determination of nodal status

For patients who underwent neck dissection, nodal status was judged by the final histopathologic report. For patients who only received biopsy or PLR, nodal status was determined by imaging study. Nodes were considered to be metastatic in the presence of necrosis or extracapsular spread [17–24]. Any lateral retropharyngeal lymph node (RLN) with a shortest axial diameter ≥ 5 mm was considered metastatic [17]. Criteria for diagnosis of the cervical nodes metastasis also include nodal size and grouping. if their shortest axial diameter was ≥ 10 mm or if there was a group of three or more contiguous and confluent lymph nodes, each of which has a maximal diameter of 8 to 15 mm [25, 26].

### Analysis of distribution of metastatic node

The actual distribution of metastatic lymph nodes were analyzed according to the updated consensus guidelines for neck node levels published in 2014 [27].
